# Integrated molecular data analysis confirms PDK1 as a candidate risk factor in ALS pathophysiology

**DOI:** 10.1186/s13041-025-01248-0

**Published:** 2025-10-01

**Authors:** Yuqian Liu, Ruiyun Guo, Ni Wang, Yue Yang, Jialu Li, Danyang Jing, Ruoyan Cui, Runchao Ma, Jun Ma

**Affiliations:** 1 Hebei Medical University-Galway University Stem Cell Research Center, Hebei Medical University, Shijiazhuang, 050017 Hebei Province China; 2 Hebei Research Center for Stem Cell Medical Translational Engineering, Hebei Medical University, Shijiazhuang, 050017 Hebei Province China; 3 Hebei Technology Innovation Center for Stem Cell and Regenerative Medicine, Hebei Province 050017 Shijiazhuang, China; 4 Hebei International Joint Research Center for Stem Cell and Regenerative Medicine, Hebei Province 050017 Shijiazhuang, China; 5 Human Anatomy Department, Hebei Medical University, Shijiazhuang, 050017 Hebei Province China

**Keywords:** Amyotrophic lateral sclerosis, PDK1, Oxidative stress, pQTL, eQTL, Transcriptome analysis, Cell transfection

## Abstract

Combining cellular, animal, and MR analyses from three independent cohorts, we identified PDK1 as a consistent risk factor for ALS development, highlighting its potential as a therapeutic target. To further elucidate PDK1’s pathogenic mechanisms, we conducted transcriptomic profiling. Samples were stratified into PDK1 high- and low-expression groups. GO and KEGG analyses demonstrated that upregulated DEGs were enriched in pathways involving β-CATENIN, cell adhesion and Ribosome, suggesting a potential role for WNT/β-catenin signaling activation in ALS pathogenesis. To further validate the consistent risk association of PDK1 with ALS across multiple datasets, we utilized 4-month-old SOD1G93A transgenic mice, 4-month-old C9orf72 transgenic mice, and SOD1-overexpressing HEK293T cells. Significant upregulation of PDK1 mRNA was observed in all models, and a significant increase in protein abundance was found in SOD1G93A. This provides strong experimental evidence for the results of the MR study. These results indicate that PDK1 may affect the pathogenesis of amyotrophic lateral sclerosis through genetic variations and transcriptional dysregulation, and may play an important role in the occurrence and development of the disease.

**Supplementary Information:**

The online version contains supplementary material available at 10.1186/s13041-025-01248-0.

## Introduction

Amyotrophic lateral sclerosis (ALS) is a fatal neurodegenerative disorder characterized by progressive motor neuron degeneration. While extensive studies implicate oxidative stress in ALS pathogenesis, the precise causal relationship between these two factors remains unclear. To date, RCTs have investigated the role of premorbid oxidative stress in ALS risk. Mendelian randomization (MR) is an analytical approach employed to assess the causal relationship between an observable, modifiable exposure or risk factor and a related outcome. This method is particularly useful when causal relationships cannot be determined in randomized controlled trials, and observational studies may produce biased associations due to confounding factors or reverse causality, which is the core basis of our research approach [5].

**Fig.1:**
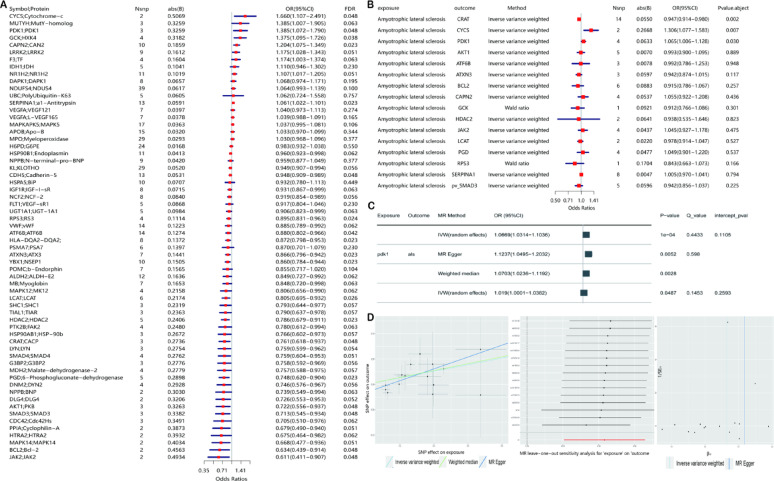
**A** Cis-pQTL Mendelian randomization analysis was performed on 1187 whole blood proteins with high correlation scores, followed by genome-wide correction. **B** A secondary correction analysis using genomic cis-eQTL was conducted on the proteins that showed significance in the proteomic analysis. Both A and B correction methods applied FDR correction to all included genes or proteins. **A** & **B**. CRAT, CYCS, and PDK1 were jointly identified as having significant promoting effects on ALS in terms of both protein abundance and gene expression. **C** The positive correlation between PDK1 and the risk of ALS was further validated using the IEU additional database. **D** Scatter plot, leave-one-out analysis, and funnel plot were used to demonstrate the causal effect of PDK1 on ALS, as shown in panel **D** MR analysis was performed using the IVW method to estimate the causal effects of oxidative stress-related proteins and their corresponding genes on ALS. Only strong instrumental variables (F-statistics > 10) were selected. The IVW approach was applied to summary statistics from multiple genetic instruments to assess the causal relationship between each protein, gene and ALS risk. The robustness of the results was further confirmed through sensitivity analyses, including IVW, MR-Egger, MR-PRESSO, and other complementary methods

**Fig. 2:**
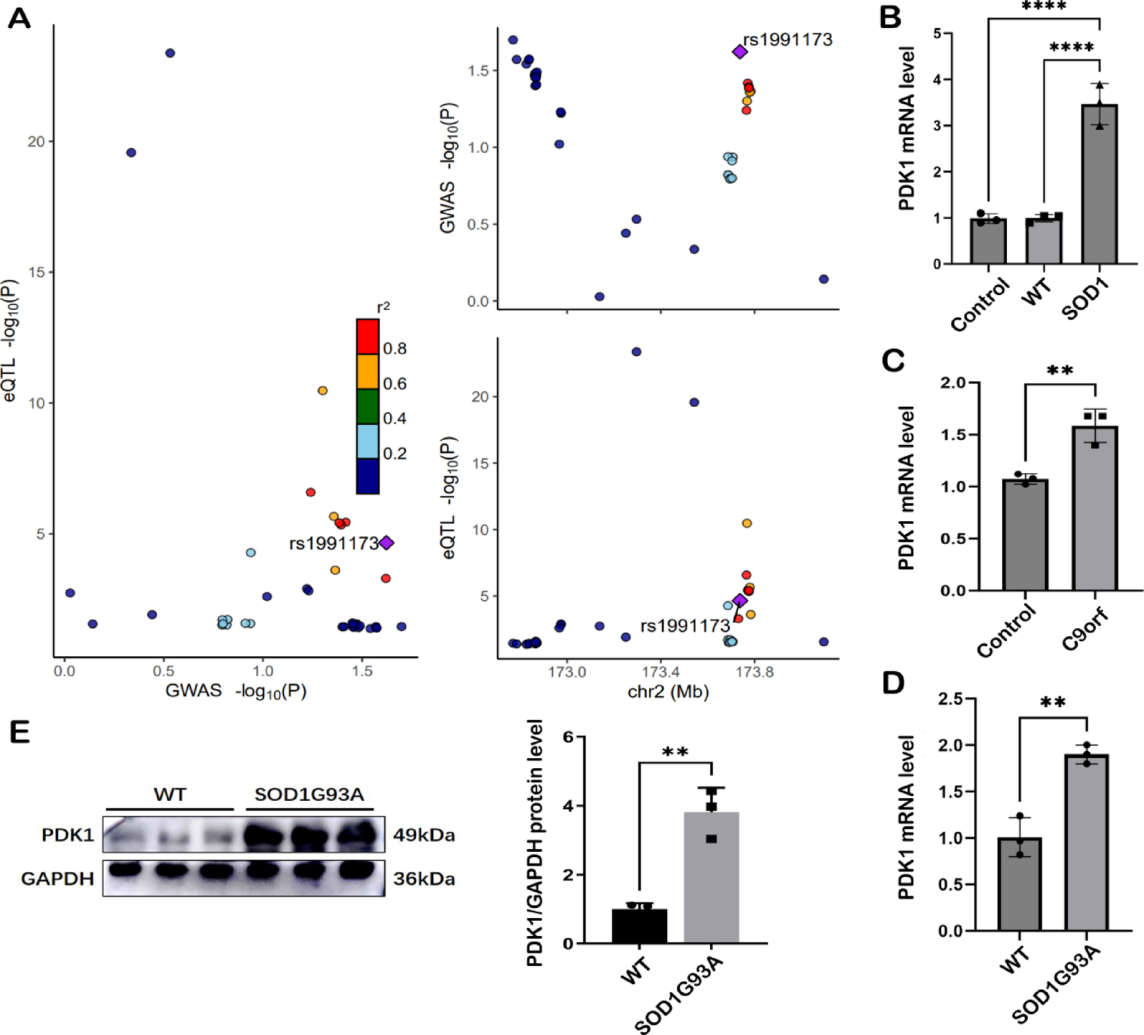
**A** The association between PDK1 single nucleotide polymorphisms (SNPs) and ALS was statistically significant at every examined point. The x-axis represents p-values from GWAS, while the y-axis denotes p-values from cis-eQTL analysis (posterior probability for shared causal variants: PDK1 = 0.9998). Regional association plots for variants within PDK1 in whole blood in relation to ALS are displayed in panel 2A. The colocalization method was applied to correct for biases due to linkage disequilibrium. With a posterior probability threshold set at 0.9, the colocalization analysis indicates a high likelihood of shared incidental variants between the oxidative stress-related gene PDK1 in whole blood and ALS. Furthermore, PDK1 corresponds to the gene identified in our MR analysis, reinforcing the evidence presented in our study. However, no colocalization evidence was found for CRAT or CYCS. **B** RT-qPCR was performed to assess mRNA expression levels in cell line models transfected with overexpression control (n = 3), blank transfection (n = 3), and SOD1 (n = 3), yielding *p* = 0.0001. (C) PDK1 mRNA expression was quantified in spinal cord tissues via RT-qPCR, with biological triplicates for both C9orf72 experimental (n = 3) and wild-type control (n = 3) groups. Statistical analysis by t-test indicated a significant increase in the C9orf72 cohort (t = 4.161, *p* = 0.0019). **D** PDK1 mRNA expression levels in spinal cord tissues were quantified using RT-qPCR, comparing SOD1-G93A transgenic mice (n = 3) with wild-type controls (n = 3). Statistical analysis by t-test revealed a significant upregulation in the SOD1-G93A group (t = 6.930, *p* = 0.0023). **E** Western blot analysis was performed to validate protein expression levels in spinal cord tissues of SOD1G93A mice (n = 3) and wild-type controls (n = 3). The results demonstrated a statistically significant upregulation of PDK1 at both protein and transcriptional levels (t = 6.657, *p*= 0.0026), the experiments indicated that there was significant evidence of upregulation of PDK1 both in protein and gene, providing evidence for genome-wide association. Colocalization analysis was performed using a Bayesian framework to assess whether oxidative stress-related genes and ALS risk share causal genetic variants. We applied the colocR package to compute posterior probabilities under different hypotheses, with a PPH4 > 0.9 indicating significant colocalization. This analysis confirmed genetic overlap between oxidative stress-related genes and ALS. **B**–**E** All statistical analyses and graphical representations were generated using GraphPad Prism10. For the cell-based assay in panel B, Brown-Forsythe test yielded a p-value > 0.05, and homogeneity of variance was met; thus, one-way ANOVA was applied. For the two-group comparisons in panels **C**, **D**, and **E**, normality was confirmed by Shapiro–Wilk test (*p* > 0.05), and homogeneity of variance was met; therefore, unpaired t-tests were used

Summary statistics of ALS were obtained from the most recent and largest ALS GWAS, which contained 10 million SNPs in 138,086 European individuals (27,205 ALS cases and 110,881controls, GCST90027164). This study utilized Cis-expression Quantitative Trait Loci (eQTL) [8] and Cis-protein Quantitative Trait Loci (pQTL) datasets [2] as discovery cohorts (data sources in Supplementary Material S2). Cis-eQTL were defined as variants within ± 1 Mb of regulated genes, and all pQTL analyzed were Cis-acting. After filtering weak instrumental variables (F-statistic > 10), we performed MR analysis on 1,189 oxidative stress-related proteins (GeneCards Relevance score > 7) [10] Our analysis identified several proteins, including MUTYH, Cyt-C, PDK1, CRAT, VWF, and JAK2, as potential contributors to ALS pathogenesis (Fig. 1A), though further validation is required to confirm their roles. To refine these findings, we leveraged Cis-eQTL data to further screen the significant proteins identified from Cis-pQTL analysis. This revealed that PDK1, Cyt-C, and CRAT also influence ALS at the genetic level (Fig. 1B). Both Cis-eQTL and Cis-pQTL results were adjusted using the FDR for all included genes or proteins to mitigate potential bias arising from multiple MR analyses. Notably, PDK1, Cyt-C, and CRAT emerged as consistent risk factors across both datasets, demonstrating significant associations with ALS. However, due to limitations in the number of instrumental variables, we validated these findings using an independent dataset from the oxidative stress-related IEU database [6] (Fig. 1C, D). While PDK1 remained significantly associated with ALS risk, no significant association was observed for Cyt-C, and CRAT could not be validated due to limited data, necessitating further investigation.In our MR analysis, the Inverse-Variance Weighted (IVW) method served as the primary analytical approach, supplemented by Weighted Median (WM), MR-Egger, and MR-PRESSO as secondary methods. The robustness of the results was confirmed through leave-one-out sensitivity analysis, and MR-PRESSO detected no significant heterogeneity or pleiotropy (*P* < 0.05). Colocalization analysis further supported PDK1-ALS causality, indicating shared genetic variants between oxidative stress-related genes in whole blood and ALS (Fig. 2A; details in Additional file1.S12). Specifically, we observed that a one standard deviation increase in PDK1 was associated with a 3.75% increase in ALS risk, as illustrated in the MR scatter plot and leave-one-out sensitivity analysis [9] (Fig. 1D, H). To ensure unidirectional causality and rule out reverse causation, we conducted reverse MR analyses across three independent cohorts. No evidence of reverse causality was detected for PDK1, reinforcing its role as a positive risk factor for ALS and effectively excluding the possibility of bidirectional effects.

To further validate the consistent risk association between PDK1 and ALS across multiple datasets, we first confirmed the expression level of PDK1 at the genetic and protein level. The detailed primer sequences, cell transfection methods, and RNA and protein extraction methods, as well as the Western blotting experimental method, can be found in Additional file 1.S13. In SOD1 overexpressed HEK293T cells, *p* = 0.0001 (Fig. 2B), in the spinal cord of 4-month-old C9orf72 transgenic mice, *p* = 0.0019 (Fig. 2C); in the spinal cord of 4-month-old SOD1 transgenic mice, *p* = 0.0023 (Fig. 2D); we found that PDK1 was significantly upregulated in all three ALS biological models. Then, we conducted a Western blot experiment at the protein level for verification, and found that the protein abundance of PDK1 in the spinal cord of 4-month-old SOD1G93A transgenic mice was also significantly upregulated, *p* = 0.0026 (Fig. 2E). Each experimental group contained three biological replicate samples, and each replicate sample was tested three times. This provided strong experimental evidence for the discovery of MR. These results indicate that PDK1 may affect the pathogenesis of ALS through genetic variations and transcriptional dysregulation, and may play an important role in the occurrence and development of the disease.

**Fig. 3:**
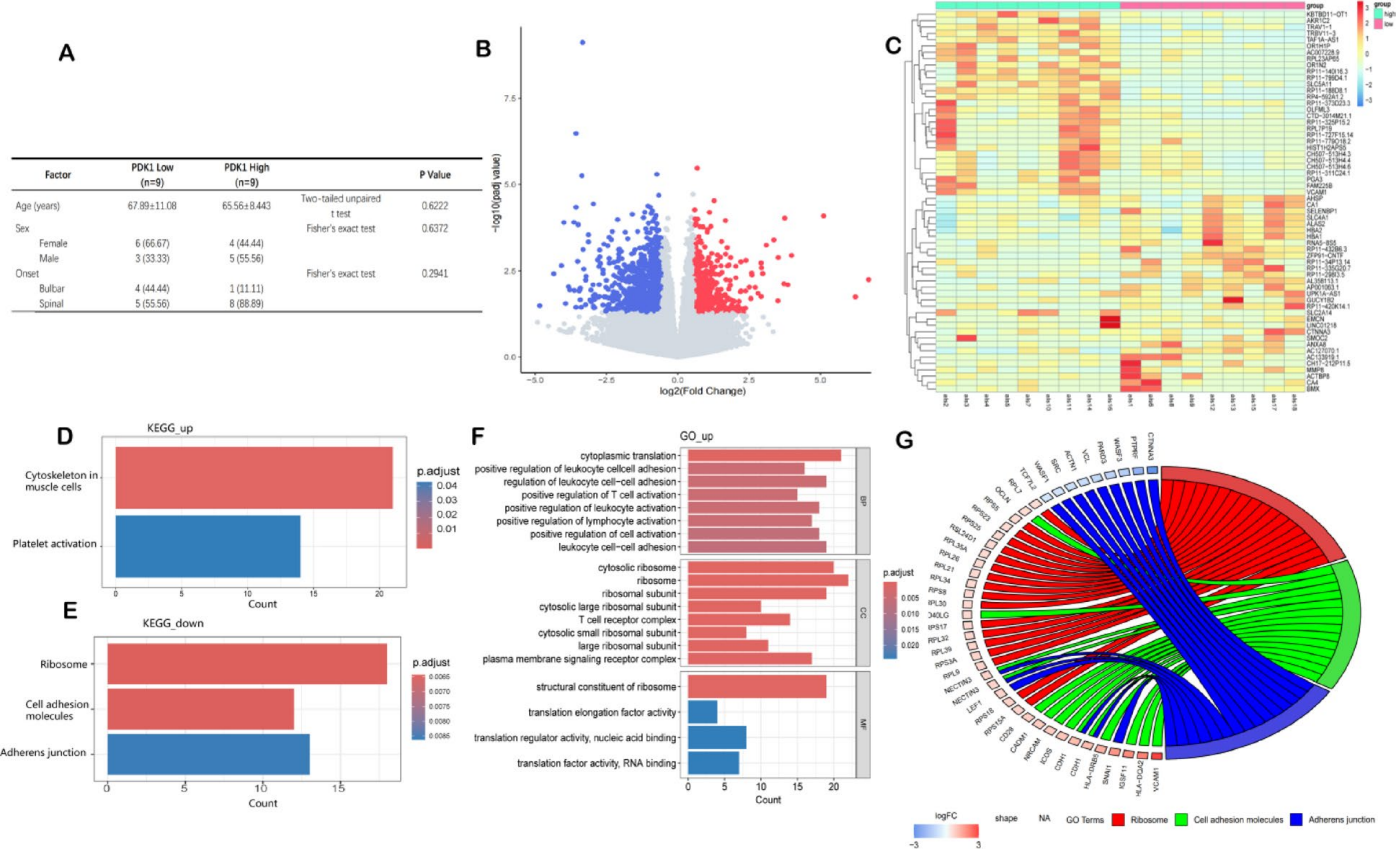
**A** To further investigate the potential mechanism of PDK1 in ALS, 18 ALS samples from the GSE183204 dataset were subgrouped by disease status. Covariates and chi-square tests were used to exclude the influence of age, sex, and disease subtype. **B** Differential gene expression analysis between PDK1 high- and low-expression groups in ALS identified 1464 differentially expressed genes (485 upregulated and 979 downregulated) with |logFC|> 0.58 and padj < 0.05. **C** Heatmaps of the two groups of differential expression analysis are shown. Blue indicates downregulated genes, while red represents upregulated genes. **D** The downregulated 979 genes were enriched in the most significant pathways, including "Cytoskeleton in muscle cells" and "Platelet activation." **E** The upregulated 485 genes were enriched in significant pathways such as "Cell adhesion molecules," "Ribosome," and "Adherens junction." **F** GO enrichment analysis of upregulated genes at the CC, BP, and MF levels revealed potential microscopic mechanisms. **G** KEGG pathway analysis of upregulated genes highlighted significant pathways that may influence the development and progression of ALS. The potential mechanisms were preliminarily screened using RNA-sequencing data to compare the gene expression patterns between two groups with high and low PDK1 expression levels. This analysis utilized edgeR. After normalizing and standardizing the data, it conducted the analysis of differential genes (adjp < 0.05,|logFC|> 0.58). Subsequently, GO and KEGG pathway enrichment analyses were carried out using the clusterProfiler R package to identify significantly enriched biological processes and pathways

Combining cellular, animal, and MR analyses from three independent cohorts, we identified PDK1 as a consistent risk factor for ALS development, highlighting its potential as a therapeutic target. To further elucidate PDK1’s pathogenic mechanisms, we conducted transcriptomic profiling. This study focuses on sporadic ALS (sALS, accounting for 90%-95% of ALS cases), due to its higher clinical representativeness and unclarified genetic heterogeneity. After stratifying patients by PDK1 expression levels, although there was no significant difference in age between the two groups (supplementary material or Fig. 3A), the high PDK1 group showed a trend of earlier onset age, suggesting that PDK1 may be related to the early onset of the disease. Since current research on PDK1 in ALS is scarce, this study aims to explore the potential pathway mechanisms of sALS, and the increasing expression trend of PDK1 may provide new clues for disease progression. Potential confounding effects of age and sex were addressed using chi-square tests (Additional file 1.S14) and covariate adjustments (Fig. 3A), with no significant confounding detected. Samples were stratified into PDK1 high-expression and low-expression groups, revealing 1,464 DEGs, including 979 downregulated and 485 upregulated genes (adjp < 0.05, |logFC|> 0.58) (Fig. 3B, C). Gene GO and KEGG analyses (Fig. 3D–F) demonstrated that upregulated DEGs were enriched in pathways involving β-CATENIN, Ribosome and cell adhesion, suggesting a potential role for WNT/β-catenin signaling activation in ALS pathogenesis (Fig. 3G).

PDK1 is a critical node in oxidative stress and energy metabolism pathways. It regulates the oxidative respiratory chain through its interactions with pyruvate dehydrogenase (PDH), Complex I, and Complex III, which are essential for electron transport and energy production. Additionally, PDK1 functions as an effector molecule in the WNT/β-catenin signaling pathway. Activation of the canonical WNT/β-catenin pathway stimulates aerobic glycolysis by upregulating glycolytic enzymes such as PDK1 and monocarboxylate transporter 1 (MCT-1). This metabolic shift, known as the Warburg effect, plays a pivotal role in energy metabolism regulation [7]. WNT ligands bind to Frizzled (FZD) and LRP-5/6 receptors, triggering phosphorylation and activation of the Axin/APC complex. This process inhibits glycogen synthase kinase-3 beta (GSK-3β) mediated phosphorylation of β-catenin, preventing its proteasomal degradation. Consequently, β-catenin accumulates in the cytoplasm and translocates to the nucleus, where it activates the transcription of glycolytic enzymes, which are critical for cellular metabolism [3]. Prior to nuclear translocation, β-catenin gradually accumulates in the cytoplasm and interacts with the cytoplasmic domains of cadherin and actin-binding proteins, regulating cadherin aggregation [1]. This interaction disrupts cellular connectivity, tissue structural integrity, and homeostasis, contributing to disease pathogenesis.In ALS, PDK1 dysregulation not only impairs energy metabolism by suppressing the TCA cycle and glycolysis—leading to reduced oxidative phosphorylation and ATP production—but also plays a significant role in the WNT/β-catenin pathway. Transcriptomic analysis revealed PDK1’s involvement in cell adhesion processes (KEGG pathway hsa04520), where β-catenin acts as a core mediator of adhesive connections. Dysregulation of this pathway disrupts cell-to-cell communication, tissue integrity, and homeostasis, potentially exacerbating ALS pathology. PDK1 also directly modulates the balance between mitochondrial oxidative phosphorylation and glycolysis through its regulation of the pyruvate dehydrogenase complex (PDC). PDC converts pyruvate into acetyl-CoA, which enters the TCA cycle for oxidative phosphorylation. In ALS patients, elevated PDK1 expression may inhibit PDC activity, diverting pyruvate to cytoplasmic glycolysis. Lactate dehydrogenase (LDH) then metabolizes pyruvate into lactate, which accumulates in neurons, enhancing mitochondrial activity and promoting oxidative stress. Concurrently, PDK1 dysregulation demonstrates significant associations with ribosomal biogenesis and translational pathways (Fig. 3N). While PDK1-mediated inhibition of PDH reduces acetyl-CoA production, enhanced glycolysis may compensate through ATP-citrate lyase (ACLY)-generated acetyl-CoA, thereby promoting histone acetylation of ribosomal genes [11]. This metabolic shift simultaneously augments glycolysis to increase nucleotide and amino acid availability, supporting rRNA transcription and ribosomal assembly [12]. Furthermore, PDK1 upregulation leads to lactate accumulation, which inhibits histone deacetylases (HDACs) to enhance ribosomal protein (RPs) gene expression while concomitantly providing energy for translation elongation [13].

These findings align with prior experimental studies, which have demonstrated that the pyruvate dehydrogenase kinase (PDK) inhibitor dichloroacetate (DCA) exerts multidimensional neuroprotection in the SOD1G93A rat model of ALS [4].

Key observations include: Firstly, Metabolic reprogramming: DCA restores tricarboxylic acid cycle activity (TCA), corrects a “Warburg-like” metabolic phenotype, and enhances glucose oxidation (The activity of the TCA cycle has increased by approximately two times.). This metabolic shift may converge with our enriched pathways by promoting histone acetylation of ribosomal genes; improved metabolic homeostasis simultaneously supports the ATP and amino-acid supply required for translational fidelity. Secondly, Neuroprotection: Motor-neuron survival is increased, and neuroinflammation is attenuated (IL-6 levels fall by approximately 60%). Thirdly, Functional benefit: Motor decline is delayed, as shown by approximately 2.1-fold improvement in Rotarod performance. Fourthly, Survival advantage: Median lifespan is extended by approximately 19%. Collectively, DCA has been shown to promote weight gain, restore mitochondrial gene expression, enhance muscle strength, attenuate neuronal toxicity, reduce motor-neuron loss and glial accumulation, and improve both motor performance and survival in SOD1G93A rats, with similar benefits reported in ALS mice.

Emerging evidence further indicates that PDK1-mediated metabolic reprogramming antedates the onset of overt motor symptoms; skeletal-muscle glucose metabolism shifts toward lipid utilization in pre-symptomatic disease [14]. These data implicate PDK1 dysregulation as an early, cross-cell-type event in ALS—spanning neurons, glia, and muscle—and underscore its potential causal role. Our study was designed to identify exposures causally linked to disease initiation, thereby uncovering novel therapeutic targets and risk factors. This is in line with our research, which aims to identify the exposure factors that have a causal relationship with the disease before it occurs, in order to discover new therapeutic targets and the factors that may cause the disease. This might be related to the early dysregulation of PDK1 and the pathogenesis of ALS. However, the causal relationships between specific stages of disease occurrence and development still require further investigation. Based on the existing evidence chain, it is suggested to develop a phased strategy to optimize the DCA administration plan and intervene at an earlier time point. Develop brain-targeted PDK1 to penetrate the blood–brain barrier. This will be an important translational basis. Together with our research, these indicate that the dysregulation of PDK1 is an early event in ALS across neuronal-glia-muscle cell types, and the metabolic reprogramming mediated by it occurs before the appearance of motor symptoms. The effect of early and faster intervention may be different, and it is worth further in-depth study.

## Supplementary Information

Below is the link to the electronic supplementary material.

Supplementary Material 1

Supplementary Material 2

Supplementary Material 3

## Data Availability

No datasets were generated or analysed during the current study.

## References

[1] Buechel D, Sugiyama N, et al. Parsing β-catenin’s cell adhesion and Wnt signaling functions in malignant mammary tumor progression. Proc Natl Acad Sci U S A. 2021. 10.1073/pnas.2020227118. 34408016 10.1073/pnas.2020227118 PMC8403962

[2] Ferkingstad E, Sulem P, et al. Large-scale integration of the plasma proteome with genetics and disease. Nat Genet. 2021;53(12):1712–21. 34857953 10.1038/s41588-021-00978-w

[3] Jiang X, Guan Y, et al. Potential roles of the WNT signaling pathway in amyotrophic lateral sclerosis. Cells. 2021. 10.3390/cells10040839. 33917816 10.3390/cells10040839 PMC8068170

[4] Martínez-Palma L, Miquel E, et al. Mitochondrial modulation by dichloroacetate reduces toxicity of aberrant glial cells and gliosis in the SOD1G93A rat model of amyotrophic lateral sclerosis. Neurotherapeutics. 2019;16(1):203–15. 30159850 10.1007/s13311-018-0659-7 PMC6361051

[5] Skrivankova VW, Richmond RC, et al. Strengthening the reporting of observational studies in epidemiology using Mendelian randomization: the STROBE-MR statement. JAMA. 2021;326(16):1614–21. 34698778 10.1001/jama.2021.18236

[6] Sun BB, Maranville JC, et al. Genomic atlas of the human plasma proteome. Nature. 2018;558(7708):73–9. 29875488 10.1038/s41586-018-0175-2 PMC6697541

[7] Vallée A, Guillevin R, et al. Vasculogenesis and angiogenesis initiation under normoxic conditions through Wnt/β-catenin pathway in gliomas. Rev Neurosci. 2018;29(1):71–91. 28822229 10.1515/revneuro-2017-0032

[8] Võsa U, Claringbould A, et al. Large-scale cis- and trans-eQTL analyses identify thousands of genetic loci and polygenic scores that regulate blood gene expression. Nat Genet. 2021;53(9):1300–10. 34475573 10.1038/s41588-021-00913-z PMC8432599

[9] Wu F, Huang Y, et al. Mendelian randomization study of inflammatory bowel disease and bone mineral density. BMC Med. 2020;18(1):312. 33167994 10.1186/s12916-020-01778-5 PMC7654011

[10] Xu S, Li X, et al. Oxidative stress gene expression, DNA methylation, and gut microbiota interaction trigger Crohn’s disease: a multi-omics Mendelian randomization study. BMC Med. 2023;21(1):179. 37170220 10.1186/s12916-023-02878-8 PMC10173549

[11] Wellen KE, Hatzivassiliou G, Sachdeva UM, Bui TV, Cross JR, Thompson CB. ATP-citrate lyase links cellular metabolism to histone acetylation. Science. 2009;324(5930):1076–80. 19461003 10.1126/science.1164097 PMC2746744

[12] Locasale JW, Cantley LC. Metabolic flux and the regulation of mammalian cell growth. Cell Metab. 2011;14(4):443–51. 21982705 10.1016/j.cmet.2011.07.014 PMC3196640

[13] Vander HM, Cantley LC, Thompson CB. Understanding the Warburg effect: the metabolic requirements of cell proliferation. Science. 2009;324(5930):1029–33. 19460998 10.1126/science.1160809 PMC2849637

[14] Palamiuc L, Schlagowski A, Ngo ST, Vernay A, Dirrig-Grosch S, Henriques A, et al. A metabolic switch toward lipid use in glycolytic muscle is an early pathologic event in a mouse model of amyotrophic lateral sclerosis. Embo Mol Med. 2015;7(5):526–46. 25820275 10.15252/emmm.201404433 PMC4492815

